# Pharmacists’ experiences on adverse drug reaction: 10 years later

**DOI:** 10.3389/fphar.2022.932942

**Published:** 2022-09-29

**Authors:** Mahmathi Karuppannan, Nur Azzrin Nisha Mohamad Rizal, Kok-Thong Wong, Salmiah Mohd. Ali, Kang-Nee Ting, Helen Boardman

**Affiliations:** ^1^ Department of Pharmacy Practice, Faculty of Pharmacy, Universiti Teknologi MARA (UiTM) Cawangan Selangor, Puncak Alam Campus, Shah Alam, Malaysia; ^2^ Hospital Sultanah Nora Ismail, Batu Pahat, Malaysia; ^3^ School of Pharmacy, University of Nottingham Malaysia, Semenyih, Malaysia; ^4^ Asian Metropolitan University, Kuala Lumpur, Malaysia; ^5^ Division of Pharmacy Practice and Policy, School of Pharmacy, University of Nottingham, Nottingham, United Kingdom.

**Keywords:** adverse drug reaction, pharmacists, community pharmacists, reporting ADRs, survey

## Abstract

Adverse drug reaction (ADR) is one of the leading public health concerns associated with high mortality rate. Healthcare professionals, particularly pharmacists, have a significant role in monitoring and preventing ADRs. This study was conducted on Malaysian Pharmaceutical Society (MPS) pharmacists who worked at the hospitals, health clinics, and community pharmacies to determine if pharmacists’ experiences on ADRs are still the same 10 years later. In 2010, a postal survey and in 2020, an online survey were conducted among these pharmacists. A total of 472 pharmacists and 208 participated in 2010 and 2020, respectively. About 82% and 90% of hospital/health clinic pharmacists (HCPs) observed an ADR over the last 6 months in 2010 and 2020, while 60% and 100% community pharmacists in 2010 and 2020 observed an ADR, respectively. Perindopril was the top drug (HCPs: *p* = 0.657; CPs: *p* = 0.98), and rash was the top ADR reported by the pharmacists in both years (HCPs: *p* < 0.001; CPs: *p* = 0.679). The most common actions taken by HCPs in 2010 were to report the ADR (*p* = 0.343), while in 2020, most HCPs explained to patients regarding the reaction (*p* = 0.061), which was also the same in the CP group in 2020 (*p* = 0.958). The top factor encouraging ADR reporting in both years and both pharmacist groups was the high degree of severity of the reaction (HCPs: *p* < 0.001; CPs: *p* = 0.769). While the top factors discouraging ADR reporting were a lack of information from the affected patients (HCPs: *p* = 0.2; CPs: *p* = 0.656), reaction is widely known (HCPs: *p* = 0.001; CPs: *p* = 0.144) and uncertainty of the causal relationship (HCPs: *p* = 0.169; CPs: *p* = 0.609). Majority of the pharmacists agreed that severe reactions should be reported (HCPs: *p* = 0.158; CPs: *p* = 0.501) and the main aim for reporting is to measure the incidence of ADRs (HCPs: *p* = 0.148; CPs: *p* = 0.762). Despite being able to identify ADRs during the daily practice, many pharmacists especially community pharmacists are not reporting them. There is a misconception on the purpose of reporting ADRs. An interventional program and ADR reporting training would be a useful step in improving ADR reporting practice.

## Introduction

Adverse drug reaction (ADR) is defined by the World Health Organization (WHO) as “a response to a drug which is noxious and unintended, and which occurs at doses normally used in patient for the prophylaxis, diagnosis, or therapy of disease, or for the modification of physiological function” (World Health Organization, 2020). ADR is one of the leading public health concerns associated with high morbidity and mortality rates, causing prolongation of hospitalization, unnecessary readmission, and increased healthcare expenditure (Sultana et al., 2013; Chan et al., 2016). Thus, post-marketing surveillance is essential to monitor the ADRs of new drugs in the market (Sultana et al., 2013).

Spontaneous ADR reporting is the mainstay of monitoring adverse drug reactions of newly marketed drugs. Since the thalidomide incident, WHO initiated the International Programme for Adverse Drug Reaction Monitoring for global drug safety monitoring (Olsson, 1998). Together with the United States Food and Drug Administration (FDA) and the European Medicines Agency, WHO advanced the regulatory practice protecting the global community (Olsson, 1998). Spontaneous reporting systems were first established in the Netherlands, United Kingdom, and Denmark in the 1960s. Many countries followed suit soon after.

In Malaysia, all suspected ADR cases are submitted to the Malaysian Adverse Drug Reactions Advisory Committee (MADRAC) which will then be submitted to the Uppsala Monitoring Centre in Sweden for inclusion into the WHO database (Aziz et al., 2007). Although an ADR reporting system has been in place since decades, underreporting of ADRs is still a nagging issue. A study among community pharmacists in Malaysia reported that the percentage of underreporting ADR is as high as 90–95% (Elkalmi et al., 2014).

Compared to other healthcare professionals, pharmacists are highly perceived for their role in implementing pharmacovigilance (PV) principles and reporting ADRs in their daily clinical practice as it is their core duties (Elkalmi et al., 2014; Bahnassi and Al-Harbi, 2018). Most pharmacists are aware of the existence of a system for reporting; however, only a few pharmacists have reported (Alsaleh et al., 2017). A study conducted in 2010 described that either these pharmacists lack knowledge on the process of reporting an ADR or lack confidence on which ADR to report. Identifying an ADR is challenging. Healthcare professionals sometimes fail to recognise that an ADR has occurred by misinterpreting patients’ complaints or symptoms as minor and irrelevant, or related to the progression of their medical conditions. This may explain why many ADRs are never recognized (Dormann et al., 2003). These highlight the need for a robust education in spontaneous ADR reporting systems and pharmacovigilance for pharmacists (Elkalmi et al., 2014).

This study was conducted to identify whether pharmacists were able to identify ADRs during their daily routine and the actions taken once the ADRs were identified. This survey was conducted in 2010 and 10 years later in 2020 to compare whether there were any changes in the context of practices among pharmacists in regard to identifying ADRs. The result from this research study can be used as a hallmark for stakeholders to execute a plan to develop new strategies to further improve pharmacists’ knowledge, attitude, and practices towards identifying and reporting ADRs.

## Material and methods

### Study design

This was a questionnaire-based study. The questionnaire on experiences of pharmacists on ADRs in Malaysia was administered in 2010 and 2020. The population involved all Malaysian Pharmaceutical Society (MPS)–registered pharmacists who were working at hospitals, health clinics, or community pharmacies. The study in 2010 involved a postal survey. A survey pack comprised a cover letter explaining the survey, the questionnaire, and a post-paid return envelope was mailed to all MPS-registered pharmacists. A second reminder with the survey pack was sent 2 months later. This survey was conducted between January 2010 and May 2010.

Meanwhile, in 2020, an online survey was conducted. A cover letter and the questionnaire were transferred to Google Forms and the link was sent out to all MPS pharmacists through emails and WhatsApp messages of MPS pharmacists with the help of MPS secretariats. Reminders were sent every month for about three times. This study was conducted between March 2020 and July 2020.

### Questionnaire

A questionnaire was designed following a literature search and discussions with the research team. The questionnaire was reviewed for content and validated by an expert panel of pharmacy academic and researchers (*n* = 5). A pilot study was conducted on a sample of 122 pharmacists, and the questionnaire was modified accordingly. The questionnaire was reviewed again to ensure suitability for use in 2020, and was validated by a team of experts consisting academicians and practicing pharmacists (*n* = 5).

There were four sections with a total of 21 items in the questionnaire. Section A gathered information on respondents’ demographics. Section B consisted of a screening question to know whether the respondents had direct patient contact for the last 6 months. Those who did not have a direct contact were not included in the analysis.

Section C determined respondents’ experiences on ADR:

1) whether they have observed an ADR in the last 6 months,
2) how frequently they observed an ADR in the last 6 months,
3) what type of ADRs were observed—a list of common ADRs based on reports received by the MADRAC, literature, and pilot study were created for the respondents to choose, and they could choose more than one answer. An open-ended option was also given for the respondents to fill, in case the ADR was not listed.
4) The drugs associated with the observed ADRs—a list of common drugs based on MADRAC reports, literature, and pilot study was created for respondents, and they could choose more than one answer. An open-ended option was also given for the respondents to fill, in case the drug was not listed.
5) Actions taken regarding the observed ADR—a list of actions was created based on the literature and pilot study, and the respondents could choose more than one answer. An open-ended option was also given for the respondents to fill in case the action was not listed.

Section D evaluated respondents’ attitude and awareness on reporting ADRs—whether they were aware of the available system, its aims, and the types of ADRs that should be reported and factors encouraging and discouraging ADR reporting.

Sample size was calculated using the Raosoft® Sample Size Calculator. In 2010, there were approximately 2,000 MPS-registered pharmacists in Malaysia. With a confidence level of 95%, a margin of error of 5%, and response of distribution of 50%, the calculated sample size was 323. In 2020, there were a total of 5,000 MPS-registered pharmacists in Malaysia. With a confidence level of 95%, a margin of error of 5%, and response of distribution of 50%, the calculated sample size was 357.

### Inclusion and exclusion criteria

Pharmacists registered with MPS and who worked at hospitals, health clinics, or community pharmacies were included in this study. Pharmacists who do not have any contact with patients for the past 6 months, for example, pharmacists who were working in a hospital pharmacy store or enforcement unit were excluded. This was identified through a screening question at the beginning of the questionnaire: “During your daily activities, do you have a direct contact with patients?”

### Ethical approval

Ethical approval was obtained from the Division of Social Research in Medicines and Health, School of Pharmacy, University of Nottingham, United Kingdom, and permission for conducting the survey, from the President of MPS in 2010. This research also obtained an ethical approval from the Research Ethics Committee of Universiti Teknologi MARA [600-FF (PT.5/1)], and permission to conduct online survey, from the President of MPS in 2020.

### Data analysis

Statistical analysis was performed using IBM SPSS Statistics version 20.0, and the level of significance was set at *p* < 0.05. Descriptive statistics were performed on all data. To ensure the data were entered accurately and completely, frequencies of variables were computed and checked for values outside possible ranges. The Pearson chi-square test was used to compare pharmacists’ experiences on ADR between the 2 years and between the pharmacist groups.

## Results

### Demographic data

In 2010, a total of 1,477 questionnaires were mailed to MPS-registered pharmacists. Of these, a total of 472 questionnaires were returned giving a response rate of 32%, and 438 were included in the analysis (34 were excluded because of missing data). However, the number of respondents was higher than that of the calculated sample size (*n* = 323). While in 2020, emails were sent to approximately 5,000 MPS pharmacists, and a total of 208 pharmacists responded to the questionnaire (58% of the calculated sample size and response rate of 4%).

Based on Table 1, a total of 257 hospital/clinic pharmacists (HCPs) and 181 community pharmacists (CPs) completed the questionnaire in 2010, whilst in 2020, 185 HCPs and 23 CPs completed the online survey. Generally, there was no significance difference in the demographics of the respondents (*p* = 0.17) between both years. In both years, the highest respondents were HCPs (59% and 89%) and female (74% and 76%). In 2020, 55% of the pharmacists had been in practice for 5 years and less, whereas in 2010, most of the pharmacists had more than 5 years of experiences (53%).

**TABLE 1:** Demographic data of pharmacists in 2010 and 2020.

Demographic/year	2010 (*N* = 438) n (%)		2020 (*N* = 208) n (%)	
Gender				
Male	112 (26)		53 (26)	
Female	326 (74)		155 (76)	

Level of education				
Bachelor’s degree	380 (86)		164 (79)	
Master’s degree	55 (13)		44 (21)	
Doctor of philosophy	3 (1)		-	
		
Work setting				
Hospital/health clinic	257 (59)		185 (89)	
Community pharmacy	181 (41)		23 (11)	
		
Years of work experience				
5 years or less	204 (47)		115 (55)	
More than 5 years	234 (53)		93 (45)	
	HCP	CP	HCP	CP
	*N* = 259	*N* = 182	*N* = 185	*N* = 23
Had patient contact; n (%)	226 (87)	180 (99)	152 (82)	20 (87)
	HCP	CP	HCP	CP
	*N* = 226	*N* = 180	*N* = 152	*N* = 20
Observed a suspected ADR last 6 months; n (%)	186 (82)	107 (60)	137 (90)	20 (100)
	HCP	CP	HCP	CP
	*N* = 186	*N* = 107	*N* = 137	*N* = 20
Reported an ADR before; n (%)	163 (88)	15 (14)	110 (81)	8 (40)

Of the 185 HCPs in 2020, 152 (82%) claimed to have direct contact with patients in the past 6 months, while 20 CPs (87%) out of 23 claimed the same. In 2010, 87% (n = 226) out of 259 HCPs, and 99% of CPs claimed to have direct contact with patients. Pharmacists who did not have any contact with patients for the past 6 months were excluded from further analysis.

### Pharmacists’ experiences on ADRs

Pharmacists were asked to state their experiences of observing ADRs in the last 6 months (Table 1). In 2020, 90% of 152 HCPs and 100% CPs encountered an ADR in the last 6 months. In 2010, 82% of 226 HCPs and 60% of 180 CPs reported the same. Pharmacists who did not encounter any ADR in the last 6 months were excluded from further analysis. Of the pharmacists who have encountered an ADR in the last 6 months, 88% and 81% of HCPs in 2010 and 2020 have reported an ADR before, respectively, while only 14% and 40% of CPs in 2010 and 2020 have done so, respectively.

Table 2 shows the comparison of responses between HCPs and CPs in 2010. The top five adverse drug reactions encountered by pharmacists in 2010 were rash, itchiness, dry cough, dizziness, and headache. Only dry cough showed significant difference between the two pharmacist groups. However, rash was the most reported ADRs in both groups. The top five drugs associated with the observed ADRs in 2010 were perindopril, aspirin, metformin, diclofenac, and amlodipine. There were significance differences between HCPs and CPs for all drugs except diclofenac, while perindopril was on the top of the list in both groups. Among the top five actions taken regarding the observed ADRs, only “make note in patient’s chart” had no significance difference between HCPs and CPs. It is also important to note that only 1% of CPs in 2010 reported the ADRs but more than 60% asked patients to inform doctors and explained to patients regarding the reaction.

**TABLE 2:** Comparison between hospital/health clinic pharmacists (HCPs) and community pharmacists (CPs) in 2010.

	HCP (*n* = 186)	CP (%) (*n* = 107)	^a^ (%)*p*-value
Types of ADRs observed (top five in 2010)			
Rash	52	60	0.175
Itchiness	44	54	0.079
Dry cough	31	46	0.012
Dizziness	32	35	0.616
Headache	31	24	0.210

Drugs associated with the observed ADRs (top five in 2010)			
Perindopril	37	42	0.011
Aspirin	17	28	<0.001
Metformin	11	34	<0.001
Diclofenac	20	15	0.869
Amlodipine	16	25	0.001

Actions taken regarding the observed ADRs (top five in 2010)			
Explain to patient regarding the reaction	48	61	<0.001
Ask patient to inform doctor	44	64	<0.001
Send report to MADRAC	52	1	<0.001
Suggest to patient to stop the medicine	20	43	<0.001
Make note in patient’s chart	32	25	0.992

The type of ADRs pharmacists believe should be reported (top five in 2010)			
Severe reactions	97	75	0.816
Reactions to new drugs	95	61	<0.001
Unexpected/unusual reactions	92	63	0.011
Certain (sure, ascertained) reactions	87	58	0.005
Teratogenicity phenomenon	86	51	<0.001

Factors encouraging pharmacists to report a suspected ADR (top five in 2010)			
The high degree of severity of a clinical reaction	96	70	0.077
The involvement of a newly licensed drug	86	50	<0.001
The reaction is not widely known	82	47	<0.001
The specific typology of the reaction (unusual/unexpected)	81	45	<0.001
An obvious causal relationship with the administration of the drug	81	42	<0.001

Factors discouraging pharmacists to report an ADR (top five in 2010)			
Lack of information from the affected patient	74	55	<0.001
The uncertainty of a causal relationship with the administration of the drug	60	55	<0.001
The reaction is widely known	52	62	<0.001
The uncertainty of the type of reactions to be reported	57	52	0.004
The low degree of severity of a clinical reaction	45	53	<0.001

Pharmacists believes on the aim of monitoring the spontaneous reporting of suspected ADRs (top five in 2010)			
To measure the incidence of ADRs	94	74	0.597
To identify uncommon ADRs	97	68	0.007
To identify previously unknown ADRs	94	66	0.112
To maintain a database of ADRs	88	67	0.554
To identify factors predisposing patients to ADRs	78	58	0.072

a Pearson chi-square.

Table 3 shows the comparison of responses between HCPs and CPs in 2020. Of the top five ADRs encountered by pharmacists in 2020, only dry cough showed significant difference between HCPs and CPs which is similar to 2010 data. However, a new ADR, oedema periorbital, appeared in top five list, whereas dizziness was not in the top five list as in 2010. The top five drugs associated with the observed ADRs in 2020 were perindopril, diclofenac, amoxicillin, amlodipine, and mefenamic acid. This list was different from the list in 2010 where aspirin and metformin were replaced with amoxicillin and mefenamic acid. All drugs showed no significant difference between HCPs and CPs. However, it is worth noting that perindopril was the highest drug reported in both years. The action “explain to patient regarding the reaction” topped the list in 2010 and 2020. Although this action had significance difference in 2010, it was found statistically not different in 2020. Only the actions “send report to drug information center,” “make note in patient’s chart,” and “send report to MADRAC” were statistically different in both pharmacist groups in 2020.

**TABLE 3:** Comparison between hospital/health clinic pharmacists (HCPs) and community pharmacists (CPs) in 2020.

	HCP (*n* = 137)	CP (*n* = 20)	^a^ *p*-value
Types of ADRs observed (top five in 2020)			
Rash	76%	35%	0.083
Itchiness	72%	9%	0.816
Oedema periorbital	38%	5%	0.971
Dry cough	28%	40%	0.014
Headache	28%	5%	0.115

Drugs associated with the observed ADRs (top five in 2020)			
Perindopril	26%	35%	0.179
Diclofenac	23%	35%	0.101
Amoxicillin	18%	10%	0.447
Amlodipine	16%	25%	0.155
Mefenamic acid	16%	15%	0.922

Actions taken regarding the observed ADRs (top five in 2020)			
Explain to patient regarding the reaction	58%	50%	0.192
Do further evaluation	58%	45%	0.447
Send report to hospital drug information center	57%	15%	0.019
Make note in patient’s chart	54%	15%	0.033
Send report to MADRAC	52%	5%	0.002

The types of ADRs pharmacists believe should be reported (top five in 2020)			
Severe reactions	93%	60%	0.877
Reactions to new drugs	93%	55%	0.304
Unexpected/unusual reactions	91%	55%	0.433
Teratogenicity phenomenon	87%	55%	0.82
Reactions to vaccines	86%	50%	0.37

Factors encouraging pharmacists to report a suspected ADR (top five in 2020)			
The high degree of severity of a clinical reaction	92%	85%	0.588
An obvious causal relationship with the administration of the drug	75%	52%	<0.001
The involvement of a newly licensed drug	70%	60%	0.218
The reaction is not widely known	68%	54%	0.021
The specific typology of the reaction (unusual/unexpected)	67%	54%	0.04

Factors discouraging pharmacists to report an ADR (top five in 2020)			
A lack of information from the affected patient	80%	50%	0.772
The uncertainty of the type of reactions to be reported	55%	45%	0.315
The uncertainty of a causal relationship with the administration of the drug	53%	50%	0.092
The low degree of severity of a clinical reaction	34%	50%	0.002
A lack of time to report reactions	29%	50%	<0.001

Pharmacists believes on the aim of monitoring the spontaneous reporting of suspected ADRs (top five in 2020)			
To measure the incidence of ADRs	97%	60%	0.36
To identify uncommon ADRs	96%	60%	0.477
To maintain a database of ADRs	94%	60%	0.788
To identify previously unknown ADRs	93%	60%	0.959
To identify factors predisposing patients to ADRs	93%	55%	0.244

a Pearson chi-square; MADRAC, Malaysian ADR Advisory Committee.

Table 4 shows the comparison of responses of HCPs in 2010 and 2020. The top five ADRs encountered by HCPs were rash, itchiness, dry cough, headache, and dizziness. Only ADRs involving the dermatology system, rash, and itchiness were found to be statistically different and highest in both years. Among the top five drugs associated with the observed ADRs, only diclofenac and mefenamic acid had significance difference in both years in which less than 15% of HCPs in 2010 stated these drugs. However, perindopril remained in the top. Regarding the action taken, only “do further evaluation” had significant difference in both years. “Send report to MADRAC” remained in the top of the list of HCPs in 2010, whereas “explain to patient regarding the reaction” was the top in 2020.

**TABLE 4:** Comparison between hospital/health clinic pharmacists (HCPs) in 2010 and 2020.

	2010 (*n* = 186)	2020 (*n* = 137)	^a^ *p*-value
Types of ADRs observed (top five among HCPs)			
Rash	52%	76%	<0.001
Itchiness	44%	72%	<0.001
Dry cough	31%	29%	0.559
Headache	31%	28%	0.503
Dizziness	32%	25%	0.176

Drugs associated with the observed ADRs (top five among HCPs)			
Perindopril	37%	35%	0.657
Amlodipine	20%	21%	0.872
Diclofenac	11%	31%	0.001
Aspirin	17%	18%	0.841
Mefenamic acid	7%	22%	0.001

Actions taken regarding the observed ADRs (top five among HCPs)			
Send report to MADRAC	52%	57%	0.343
Explain to patient regarding the reaction	48%	58%	0.061
Send report to hospital drug information centre	43%	52%	0.117
Ask patient to inform the doctor	44%	42%	0.754
Do further evaluation	32%	58%	<0.001

The types of ADRs pharmacists believe should be reported (top five among HCPs)			
Severe reactions	97%	93%	0.158
Reactions to new drugs	95%	93%	0.353
Unexpected/unusual reactions	92%	91%	0.687
Certain (sure/ascertained) reactions	87%	91%	0.242
Reactions to vaccines	88%	86%	0.586

Factors encouraging pharmacists to report a suspected ADR (top five among HCPs)			
The high degree of severity of a clinical reaction	96%	85%	<0.001
An obvious causal relationship with the administration of the drug	81%	68%	0.009
The involvement of a newly licensed drug	86%	49%	0.001
The reaction is not widely known	82%	50%	0.001
The specific typology of the reaction (unusual/unexpected)	81%	48%	0.001

Factors discouraging pharmacists to report an ADR (top five among HCPs)			
A lack of information from the affected patient	74%	80%	0.2
The uncertainty of a causal relationship with the administration of the drug	60%	53%	0.169
The uncertainty of the type of reactions to be reported	57%	55%	0.688
The reaction is widely known	52%	28%	0.001
The low degree of severity of a clinical reaction	45%	34%	0.036

Pharmacists believes on the aims of monitoring the spontaneous reporting of suspected ADRs (top five among HCPs)			
To identify uncommon ADRs	97%	96%	0.836
To measure the incidence of ADRs	94%	97%	0.148
To identify previously unknown ADRs	94%	93%	0.765
To maintain a database of ADRs	88%	94%	0.067
To identify factors predisposing patients to ADRs	78%	93%	0.001

a Pearson chi-square; MADRAC, Malaysian ADR Advisory Committee.

Table 5 shows the comparison of responses of CPs in 2010 and 2020. Rash was the highest in 2010, whereas itchiness was the highest ADR observed in 2020. However, none of the top five ADRs had significant differences in both years. In both years, perindopril remained the top reported drug which did not have a significant difference. Only aspirin had a significant difference, where 5% CPs in 2020 and 38% CPs in 2010 reported the drug. The drug list of CPs appeared to be the same as the top five drugs list of HCPs, but the ranking differed. The most action taken by CPs in 2010 was “ask patient to inform the doctor,” whereas in 2020, it was “explain to patient regarding the action.” However, none of the actions were found to be significantly different.

**TABLE 5:** Comparison between community pharmacists (CPs) in 2010 and 2020.

	2010 (*n* = 107)	2020 (*n* = 20)	^a^ *p*-value
Types of ADRs observed (top five among CPs)			
Rash	60%	35%	0.679
Itchiness	54%	45%	0.303
Dry cough	46%	40%	0.283
Gastritis	37%	15%	0.310
Dizziness	36%	20%	0.784

Drugs associated with the observed ADRs (top five among CPs)			
Perindopril	54%	35%	0.98
Diclofenac	43%	35%	0.457
Aspirin	36%	5%	0.043
Mefenamic acid	28%	15%	0.705
Amlodipine	20%	25%	0.12

Actions taken regarding the observed ADRs (top five among CPs)			
Ask patient to inform the doctor	81%	40%	0.097
Explain to patient regarding the reaction	78%	50%	0.958
Suggest to patient a different drug	38%	25%	0.992
Make note in patient’s chart	32%	15%	0.521
Do further evaluation	25%	45%	0.001

The types of ADRs pharmacists believe should be reported (top five among CPs)			
Severe reactions	96%	60%	0.501
Unexpected/unusual reactions	80%	55%	0.714
Reactions to new drugs	79%	55%	0.608
Certain (sure/ascertained) reactions	65%	60%	0.142
Teratogenicity phenomena	65%	55%	0.163

Factors encouraging pharmacists to report a suspected ADR (top five among CPs)			
The high degree of severity of a clinical reaction	90%	60%	0.769
The involvement of a newly licensed drug	64%	40%	0.834
The reaction is not widely known	60%	20%	0.046
The specific typology of the reaction (unusual/unexpected)	58%	30%	0.418
An obvious causal relationship with the administration of the drug	54%	40%	0.616

Factors discouraging pharmacists to report an ADR (top five among CPs)			
The reaction is widely known	79%	40%	0.144
A lack of information from the affected patient	71%	50%	0.656
The uncertainty of a causal relationship with the administration of the drug	70%	50%	0.609
The low degree of severity of a clinical reaction	67%	50%	0.481
The uncertainty of the type of reactions to be reported	66%	45%	0.835

Pharmacists believes on the aims of monitoring the spontaneous reporting of suspected ADRs (top five among CPs)			
To measure the incidence of ADRs	94%	60%	0.762
To identify uncommon ADRs	87%	60%	0.579
To maintain a database of ADRs	86%	60%	0.526
To identify previously unknown ADRs	85%	60%	0.478
To identify factors predisposing patients to ADRs	75%	55%	0.433

a Pearson chi-square.

### Spontaneous ADR reporting

In 2010, the top five ADRs pharmacists believed should be reported were severe reactions, reactions to new drugs, unexpected/unusual reactions, certain reactions, and reactions of teratogenicity phenomenon (Table 2). All but “severe reactions” showed significant difference between HCPs and CPs in 2010. However, severe reactions topped the list for both the groups. In 2020, four types of ADRs in the top five list remained the same as those in 2010 (Table 3). “Reactions to vaccines” was a new addition to the list. However, all ADRs in the list had no significant difference between both groups, and “severe reactions” remained in the top of the list. When the top five list was compared with the same groups of pharmacists (Tables 4 and 5), “severe reactions” remained in the top list. Both HCPs and CPs had no significance difference on the list of ADRs to be reported when compared in both years (Tables 4 and 5).

When asked about the factors that encourage and discourage ADR reporting, the degree of severity of the reaction, whether the reaction was widely known, and the causal relationship with the administration of the drug were three of the top five factors quoted (Table 2, Table 3, Table 4, and Table 5). Other factors that encourage reporting included involvement of a newly licensed drug and specific typology of the reaction. Whilst other discouraging factors included “a lack of information from the affected patient” and “uncertainty of the type of reactions to be reported.” The factor “a lack of information from the affected patient” topped the list in 2010 and 2020. This had a significant difference between HCPs and CPs in 2010 but no significant difference in 2020.

Pharmacists were asked to identify the aims of monitoring spontaneous reporting of suspected ADRs, and the top five were “to measure incidence of ADRs,” “to identify uncommon ADRs,” “to identify previously unknown ADRs,” “to maintain a database of ADRs,” and “to identify factors predisposing patients to ADRs” (Table 2, Table 3, Table 4, and Table 5). “To measure incidence of ADRs” topped the list in both years and no significant difference was found between HCPs and CPs in 2010 and 2020 (Tables 2 and 3).

## Discussion

In 2010, 82% of HCPs and 76% CPs observed a suspected ADR, while in 2020, 90% HCPs and 100% CPs observed a suspected ADR in the last 6 months. These findings show that most pharmacists are identifying (observing) ADRs during their daily routine as quoted in a study by Irujo et al. (2007), “almost every pharmacist had detected an ADR at least once in their professional life.” Even though pharmacists are able to identify ADRs, these were not reported in most cases especially in the CP groups and similar findings were observed in another study (Alsaleh et al., 2017). Pharmacists are highly educated and have a professional responsibility in the provision of pharmaceutical care which includes the identification, prevention, and resolution of drug-related problems (DRPs). It is one of their core jobs to ensure the safe use of medicine. Reporting ADRs is equally important.

The top two types of ADRs observed by the pharmacists in this study were mostly related to the dermatological systems—rashes and itchiness. These were the same in 2010 and 2020 as well as in both HCP and CP groups. A review and a study conducted in a tertiary care hospital in India reported that ADRs related to gastrointestinal, cardiovascular, and nervous system were the most common (Geer et al., 2016; Khalil and Huang, 2020). The report by MADRAC shows that the highest number of ADR reports received was related to skin and subcutaneous tissues, and the highest number of reports received was from pharmacists (National Pharmaceutical Control Bureau, 2019). On top of that, ADR reports related to dermatology received by MADRAC have been the highest since 2010 (National Pharmaceutical Control Bureau, 2010). Skin is the most common target for ADRs. They are manifested as skin rashes and/or eruptions. Cutaneous reactions occur in 2–3% of inpatients and in about 2% of outpatients (Farshchian et al., 2015). Pharmacists can easily identify ADRs involving skin because of their objective manifestations compared to other organ systems.

The most common drug associated with the observed ADRs by both group of pharmacists in both years was perindopril. There is an increased usage of perindopril in Malaysia (Malaysian Statistics on Medicines, 2020). A study conducted in Malaysia investigating ADR-related admissions reported perindopril as one of the drugs causing the ADR-related admissions (Karuppannan et al., 2013). In another study conducted in Singapore, the most common drug category causing the ADR-related admission was cardiovascular drugs (Chan et al., 2016). Whereas in India, anti-infectives were quoted as the most common drug causing ADRs (Geer et al., 2016).

Perindopril and other angiotensin-converting enzyme (ACE) inhibitors are mostly associated with dry cough (Pinto et al., 2020). In this study, dry cough was reported as one of the most common ADRs in both years. A MADRAC newsletter reported that perindopril is the suspected drug contributing to the highest number of ADR reports, and the top three reactions associated with the drug were cough, dry cough, and dizziness (National Pharmaceutical Control Bureau, 2009). Studies were also reporting increased incidence of cough among perindopril users (Bavanandan et al., 2005), and extensive data are available on the incidence of perindopril-induced cough (Pinto et al., 2020). All these findings could have alerted healthcare professionals to be more vigilant of any signs of cough among patients who use ACE inhibitors particularly perindopril.

In response to observing ADRs, most HCPs and CPs in 2010 and 2020 claimed that they have explained to patients regarding the reaction. Delli et al. (2022) reported that through an effective interaction with patients, pharmacists are able to provide information relating to the usage of the medications, which includes the aspect of safe use of medications in order to enhance patients’ understanding and knowledge about their medications. Several studies have also reported that the most common intervention given by community pharmacists was consulting their patients regarding the drug-related problems (Schröder et al., 2011; Ylä-Rautio et al., 2020). Pharmacist and patient interactions are important to foster patient-centred care. Thus, it is a necessary skill pharmacists should acquire.

However, when comparing the actions taken between the HCP and CP groups, CPs in 2010 and 2020 were inclined to ask patients to inform their doctor regarding the ADR. This was also reported in a Spanish study of factors influencing ADR-reporting among community pharmacists, where more than 80% of CPs usually tell patients to visit their doctor when an ADR is suspected (Irujo et al., 2007). Similarly, another study conducted in Saudi Arabia claimed that approximately 77% of CPs refer patients to a doctor (Mahmoud et al., 2014). When patients report symptoms that the pharmacists attribute to potential ADRs and they think patients need to take an action, referring them to their doctor is a reasonable course of action if there is no immediate need for medical intervention.

Of the 137 CPs who claimed to have observed ADRs in 2010, only 1% have taken the action to report the ADRs to MADRAC compared to 52% out of 186 HCPs. The percentage was lower than that in a study conducted among community pharmacists in South India (12%) (Pinto et al., 2020). This is also reflected in the annual report of National Pharmaceutical Regulatory Agency (National Pharmaceutical Control Bureau, 2009). However, an increase in the number of CPs reporting was seen in 2020 (5%). Even so, this figure is still considered low compared to that of HCPs. One reason for these differences could be the types of ADRs observed by both groups of pharmacists. Minor reactions such as gastritis were more often observed by CPs and therefore, may not be reported. A few studies reported that the common reasons given by CPs for not reporting ADRs are that ADRs are not serious and already known (Irujo et al., 2007; Shaik Rahmat and Karuppannan, 2021), which is comparable to this study. Hence, pharmacists chose to solve the problem by discussing with patients (Hämmerlein et al., 2007) and most probably advise patients to stop taking the drug (Mahmoud et al., 2014).

Another reason could be that HCPs are well informed about the procedure and process of reporting ADRs (Hadi et al., 2013) compared with CPs. Previous studies have documented the lack of knowledge of CPs about ADR reporting (Hämmerlein et al., 2007; Elkalmi et al., 2014) and are mostly unsure of the types of ADRs to be reported and had insufficient knowledge on ADRs (Shaik Rahmat and Karuppannan, 2021). Thus, the CP group is prompted to refer patients to their physicians, anticipating that the physicians themselves will be able to solve and report the ADRs (Mahmoud et al., 2014). CPs may have the wrong perception that ADR reporting is the responsibility of physicians and HCPs (Mahmoud et al., 2014). In addition to educating and training CPs, perhaps it is time to remunerate pharmacists for reporting ADRs, and a study found remuneration is one of the motivating factors to report ADRs among pharmacists (Li et al., 2018).

The type of ADRs which most pharmacists in 2020 and 2010 perceived should be reported was severe reactions. Several studies reported the same—pharmacists will report if an ADR was serious or severe (Elkalmi et al., 2014; Alsaleh et al., 2017; Bahnassi and Al-Harbi, 2018; Aldryhim et al., 2019). In the study by Aldryhim et al. (2019), about 70% of pharmacists believed serious ADRs should be reported and additionally quoted that pharmacists’ therapeutic knowledge and continuous medical education were also the main factors that would encourage them to report an ADR. In Syria, 48% of the pharmacists reported seriousness of a reaction as the top in the list of factors encouraging them to report an ADR (Bahnassi and Al-Harbi, 2018).

When compared between the groups, HCPs in 2010 and 2020, “reactions to vaccines” was in the top five list. However, this was not listed in the top five list of the CP group. MADRAC, in 2015, in relation to Adverse Events Following Immunisation (AEFI), reported that there was an increment of 26.8% in the reports received relating to AEFI from the year 2014 (1,080 reports) to 2015 (1,369 reports) (Hämmerlein et al., 2007). This saw a multi-fold increase from 2020 (1,495 reports) to 2021 (28, 976 reports) presumably due to COVID-19 vaccinations (National Pharmaceutical Control Bureau, 2022). Human papillomavirus (HPV) vaccines were introduced through the HPV vaccination programme since 2010 for the prevention of cervical cancer in Malaysia (Muhamad et al., 2018). Since then, many reports relating to the vaccine were received by the MADRAC, and this accounted for a proportion as high as 87.6% (Rosli et al., 2017). This corresponds to the current findings on why the majority of pharmacists believed that reactions to vaccinations should be reported.

The WHO stated that the aims of pharmacovigilance are for early detection of previously unknown ADRs, detection of any increase in the frequency of known ADRs, identification of risk factors and possible mechanisms of ADRs, and estimation of benefit/risk analysis and dissemination of information to improve drug prescribing and regulation (World Health Organization, 2020). Based on the Malaysian Guidelines for Reporting and Monitoring (National Pharmaceutical Control Bureau, 2016), the primary purpose of reporting ADRs include an early detection of any suspected reactions, to identify uncommon drug reactions, to maintain a database for sharing ADRs information in Malaysia as well as to identify the risk factors which may predispose patients to ADRs. Most HCPs and CPs were able to identify the actual purposes. However, it was noted that at the top of the list, HCPs and CPs claimed that ADRs are reported to measure the incidence of ADRs. The incidence rate cannot be measured *via* spontaneous reporting because there is no information on the population denominator (number of people prescribed with the suspected drug). This suggests that there is a misconception in these group of pharmacists on the role of pharmacovigilance. Since reporting of ADRs is the only system which can be implemented in carrying out post-marketing surveillance in many countries including Malaysia; thus, it is crucial to improve the knowledge regarding this among pharmacists and other healthcare professional (Aziz et al., 2007; Gonzalez-Gonzalez et al., 2013) so that the significance of ADR reporting is understood and appreciated.

A lack of information from affected patients was the most cited factor discouraging reporting among HCPs in 2010. It is rather surprising since HCPs have more access to patients’ record compared to CPs, and it is reasonable if this factor was cited the highest among CPs. On top of that, this factor was still one of the top five factors in 2020 among HCPs and the percentage has increased to 80%. Similar responses were noted among pharmacists in other studies (Aziz et al., 2007; Alsaleh et al., 2017). In the process of identifying and diagnosing an ADR, it is important that detailed information is gathered from affected patients. This will guide healthcare professionals to establish a causal relationship between the reaction and the suspected drug in a reliable way (Cheema et al., 2017). It is noteworthy that “uncertainty of a causal relationship with the administration of the drugs” was also cited as one of the discouraging factors which could have been led by the lack of information from patients.

Low degree of severity of a clinical reaction, uncertainty regarding the type of reactions to be reported, and uncertainty of a causal relationship with administration of the drug remain as the major factors hindering ADR reporting similar to other studies (Edwards and Aronson, 2000; Shaik Rahmat and Karuppannan, 2021). Other factors cited were lack of training and knowledge that could have resulted in the lack of confidence in reporting ADRs (Edwards and Aronson, 2000; Alsaleh et al., 2017; Shaik Rahmat and Karuppannan, 2021). This suggested the importance of continuous training and equipping with up-to-date information regarding ADRs. A study has proven that the ADR reporting rates among pharmacists have increased up to 5.9-fold after an educational training session on pharmacovigilance (Herdeiro et al., 2008) as well as an increase in reporting of serious, unexpected, high-causality, and new drug-related ADRs (Gonzalez-Gonzalez et al., 2013).

### Limitations

The current study has several limitations. The pharmacist population in this survey may not be representative of all pharmacists in Malaysia because the experiences of non-MPS members were not explored. Members of MPS may differ from other Malaysian pharmacists in that they chose to join the professional body and thus, may be more up to date with clinical or legal issues affecting the profession. However, the extent to which being members of the MPS would have affected pharmacists’ responses is unknown.

The respondents were asked to recall the types of ADRs, causative drugs, and actions taken in response to the ADRs observed in the last 6 months. There are possibilities that pharmacists had difficulty recalling the ADRs, meaning that details may be recalled incorrectly. Furthermore, pharmacists in the hospitals or specific wards (such as medical wards or ICU) may have observed a higher number of ADRs compared with others, and it was not possible to identify this from the survey. A cross tab of the observed ADR and the drug which was responsible for the ADR could not be done because the respondents were given the choice to select more than one answer for both questions.

The online survey, although shared to all MPS pharmacists, did not reach the desired sample size of 377, and the results from this study may not represent all HCPs and CPs in Malaysia. Although measures were taken to send out the link multiple times, the survey was conducted during the peak of COVID-19 infections and announcement of lockdown in March 2020 somehow affected the number of respondents, as pharmacists were carrying out their duties to ensure continuous care was provided.

## Conclusion

This study shows that pharmacists in Malaysia encounter patients with ADRs in their daily work activities. However, there were differences in the management of patients with ADRs by hospital and community pharmacists. The role of pharmacists is important in identifying, resolving, and preventing adverse drug reactions and can be further enhanced through education and training. It is also important to emphasise the importance of reporting an ADR especially among the community pharmacists. Pharmacists also play an important role in educating patients about their drug therapy. Although the current practice of reporting ADRs by HCPs is reassuring, they should be regularly updated and reminded of the importance of reporting ADRs to ensure that this practice is continued throughout their professional life. CPs, on the other hand, should be educated about the ADR report system and understand that reporting ADRs is the responsibility of all healthcare professionals.

## Data Availability

The raw data supporting the conclusions of this article will be made available by the authors, without undue reservation.
